# A Novel Predictive Equation for Potential Diagnosis of Cholangiocarcinoma

**DOI:** 10.1371/journal.pone.0089337

**Published:** 2014-02-28

**Authors:** Ratthaphol Kraiklang, Chawalit Pairojkul, Narong Khuntikeo, Kanokwan Imtawil, Sopit Wongkham, Chaisiri Wongkham

**Affiliations:** 1 Department of Biochemistry, Faculty of Medicine, Khon Kaen University, Khon Kaen, Thailand; 2 Department of Pathology, Faculty of Medicine, Khon Kaen University, Khon Kaen, Thailand; 3 Department of Surgery, Faculty of Medicine, Khon Kaen University, Khon Kaen, Thailand; 4 Liver Fluke and Cholangiocarcinoma Research Center, Faculty of Medicine, Khon Kaen University, Khon Kaen, Thailand; National Cancer Institute, NIH, United States of America

## Abstract

Cholangiocarcinoma (CCA) is the second most common-primary liver cancer. The difficulties in diagnosis limit successful treatment of CCA. At present, histological investigation is the standard diagnosis for CCA. However, there are some poor-defined tumor tissues which cannot be definitively diagnosed by general histopathology. As molecular signatures can define molecular phenotypes related to diagnosis, prognosis, or treatment outcome, and CCA is the second most common cancer found after hepatocellularcarcinoma (HCC), the aim of this study was to develop a predictive model which differentiates CCA from HCC and normal liver tissues. An in-house PCR array containing 176 putative CCA marker genes was tested with the training set tissues of 20 CCA and 10 HCC cases. The molecular signature of CCA revealed the prominent expression of genes involved in cell adhesion and cell movement, whereas HCC showed elevated expression of genes related to cell proliferation/differentiation and metabolisms. A total of 69 genes differentially expressed in CCA and HCC were optimized statistically to formulate a diagnostic equation which distinguished CCA cases from HCC cases. Finally, a four-gene diagnostic equation (*CLDN4*, *HOXB7*, *TMSB4* and *TTR*) was formulated and then successfully validated using real-time PCR in an independent testing set of 68 CCA samples and 77 non-CCA controls. Discrimination analysis showed that a combination of these genes could be used as a diagnostic marker for CCA with better diagnostic parameters with high sensitivity and specificity than using a single gene marker or the usual serum markers (CA19-9 and CEA). This new combination marker may help physicians to identify CCA in liver tissues when the histopathology is uncertain.

## Introduction

Cholangiocarcinomas (CCA) are bile duct tumors which can be classified into intrahepatic CCA (ICC) and extrahepatic CCA. CCA is the second most common primary hepatobiliary malignancy, and its incidence is increasing globally [1]. The incidence of CCA is geographically unequal. It is high in Southeast Asia and low in Western countries [2]. The highest incidence of CCA was found in the Northeast Thailand where the liver fluke, *Opisthorchis viverrini* (Ov), is a major risk factor for CCA [3], [4]. In Western and East Asian countries, the reported risk factors are chronic inflammation and cholestatic conditions, such as primary sclerosing cholangitis, choledochal cyst, Caroli's disease, hepatolithiasis and hepatitis C infection [5]. Complete resection is the current therapy of choice. However, most cases of CCA are diagnosed at advanced stages when surgery is no longer a feasible option. The accurate interpretation of a definite diagnosis is necessary so that a medical specialist can assess the severity of the disease and select the most suitable therapy for patients. At present, histological investigation is the standard diagnosis. However, there are some biopsy specimens and poor-defined tumor tissues which cannot be definitively diagnosed by general histopathology. Hence, searching for a new diagnostic tool for these specimen is necessary.

In the past decade, many investigators have focused on the molecular and cellular perturbations which characterize the malignant phenotype. The power of a molecular signature in defining molecular phenotypes related to diagnosis, prognosis or treatment outcome was clearly seen in many studies. Several gene expression signatures have been reported for the tracking of true molecular phenotypes correlated with diseases, for example, in the classification of multiple sarcoma [6], in the outcome and chemotherapy response of ovarian cancer [7], and in the prediction of patient survival of gastric cancer [6], [8].

At present, the availability of a rapid and formal proof of malignancy is still a constant goal in the diagnosis of CCA. In the current study, we sought to develop and validate a predictive model which can differentiate tumor mass commonly found in liver, ICC and hilar CCA with liver mass from HCC and normal liver tissues. An in-house PCR array containing 176 putative CCA marker genes was tested with the training set tissues of 20 CCA and 10 HCC cases, and 69 differentially expressed genes were optimized statistically to formulate a four-gene diagnostic equation which could distinguish CCA cases from HCC cases. Finally, we validated this equation in an independent testing set of 68 CCA samples and 77 non-CCA controls. This equation was successfully validated with a high sensitivity and specificity.

## Materials and Methods

### Tissue Samples

Frozen and paraffin embedded liver tissue-microarrays from patients with histologically confirmed CCA, HCC and chronic liver diseases were obtained from a specimen bank of the Liver Fluke and Cholangiocarcinoma Research Center, Faculty of Medicine, Khon Kaen University, Thailand. Written informed consent was obtained from each subject, and the study protocol was approved by the Ethics Committee for Human Research, Khon Kaen University. The diagnosis of benign hepatobiliary disease was based on clinical and histological records.

Frozen tumor tissues from CCA (n = 20) and HCC (n = 10) cases were used as the training set and the expression profiles were examined using the in-house PCR array. The characteristics of the CCA and HCC patients are summarized in Table S1. The testing set comprised 68 cases of CCA, 47 cases of HCC (Table S2), 21 cases of non-cancerous liver tissues, and nine cases with chronic biliary-liver diseases which were biliary hyperplasia (n = 2), haemangioma (n = 2), cystadenoma (n = 2), chronic inflammation (n = 2) and hepatolithiasis (n = 1).

### In-house PCR array and Primer Design

An in-house PCR array with two duplicate sets of 191 genes was performed as a single training dataset in a 348-well microplate. Each set of 191 genes contained 176 CCA associated genes, five internal controls (*18S rRNA*, *ACTB*, *B2M*, *GAPDH* and *FDFT1*) and two for HCC markers (*AFP* and *GPC3*). To ensure that the majority of cells in the liver tissue tested were CCA cells, several cell-type markers were included in the array; these were genes for biliary cells (*KRT7* and *KRT19*), fibroblasts (*ACTA2* and *MME*), hepatocytes (*ALB* and *FGG*), and white blood cells (*ITGAL* and *PTPRC*). These markers were selectively expressed for each cell type based on the SAGE database (http://cgap.nci.nih.gov/SAGE). B2M of pooled cDNA from normal liver tissues, CCA, and CCA cell lines, was used as an inter-run reference gene.

All specific primers were designed using the following guidelines: 1) for a gene which has more than one transcript variant, the design of the primer was based on the conserved region, 2) the length of the primer was 18–25 bp, 3) the length of the designed PCR product was 75–200 bp, and 4) the optimal melting temperature was 55°C. The specificity of the primers was tested using Primer-BLAST [9] and the conventional PCR for a single PCR product verification.

Approximately 2 µg of total RNA was reverse transcribed to cDNA by the High Capacity cDNA Reverse Transcription Kit (Applied Biosystems, Foster City, CA) according to the manufacturer's protocol. An in-house PCR array was prepared using a Biomek® NX^P^ Laboratory Automate Workstation (Beckman Coulter, Fullerton, CA). Briefly, each primer pair was transferred into a 384-well white PCR plate. cDNA from each sample was mixed with 2× LightCycler® 480 SYBR Green I Master (Roche Applied Science, Mannheim, Germany) and then added to the plate. Real-time RT-PCR was performed using the LightCycler 480 II (Roche Diagnostics, Rotkreuz, Switzerland). All data were analyzed using the LightCycler® 480 SW 1.5 software. A combination of *B2M* and *GAPDH* were selected as the reference genes by NormFinder [10] and the geometric mean was used for normalising the quantities of mRNA species in each sample.

### Hierarchical Cluster Analysis

Unsupervised hierarchical cluster analysis was used to explore the differential gene expression between the CCA and HCC samples in the training set. The expression level of each gene after normalization was transformed into a 2^∧-dCp^ value. The unsupervised hierarchical analysis was performed using dChip software [11]. Independent t-tests were performed to identify genes whose expressions in the CCA samples were significantly different from those in the HCC samples. Only genes whose expressions were found to be different at the *P* value<0.05 level were selected to formulate an equation for differentiating CCAs from HCCs.

### Immunohistochemistry

Two tissue-microarrays, one of triplicate samples from CCA patients (n = 28) and one of quadruplicate samples from HCC patients (n = 24) were subjected to a standard immunohistochemical staining according to manufacturers' recommendations with the Envision Plus Detection Kit (Dako, Carpenteria, CA) for HOXB7, TMSB4, and TTR, and with the Histofine® Immonohistochemical staining reagent (Nichirei Biosciences Inc., Tokyo, Japan) for CLDN4. Tissue microarrays were treated with 1∶50 anti-CLDN4 (Santa cruz biotechnology, CA), 1∶100 anti-HOXB7 (Abnova, Taipei, Taiwan), 1∶20000 anti-TMSB4 (abcam, Cambridge, MA), and 1∶100 anti-TTR (Abnova, Taipei, Taiwan). Since almost all tissues had a similar positive frequency for immunoreactivity, the immunostaining was semi-quantitatively scored on the basis of intensity as: 0 = negative; 1+ = weak; 2+ = moderate; and 3+ = strong.

### Statistical analysis

To select candidate genes for verification and the formulation of a diagnostic equation, the normalized gene expression data which were differentially expressed between CCA and HCC were subjected to a multiple linear regression analysis using STATA version 8.0 (Stata Corporation, College Station, TX).

## Results

To prepare a gene set for the in-house PCR array, a search for CCA associated genes was conducted in 32 studies published between 2000 and 2009; three studies reported publicly available microarrays [12]–[14], one study reported SAGE data [15], and another an expression sequence tag [16]. Sixtheen of twenty upregulated genes reported in microarray database obtained from Thai patients [13] were included in this study and finally, a total of 176 genes retrieved from 1,154 reported CCA cases were selected for an in-house PCR array. As advanced hilar CCA always invade the liver parenchyma and form large focal liver mass similar to HCC, therefore the hilar extrahepatic cholangiocarcinoma with liver mass were included in this study. In CCA cases, all tested samples exhibited a high expression of biliary cell markers and a low expression of hepatocyte markers; the reverse occurred for the for HCC specimens.

### Unsupervised Hierarchical Clustering of CCA and HCC

**Figure 1:**
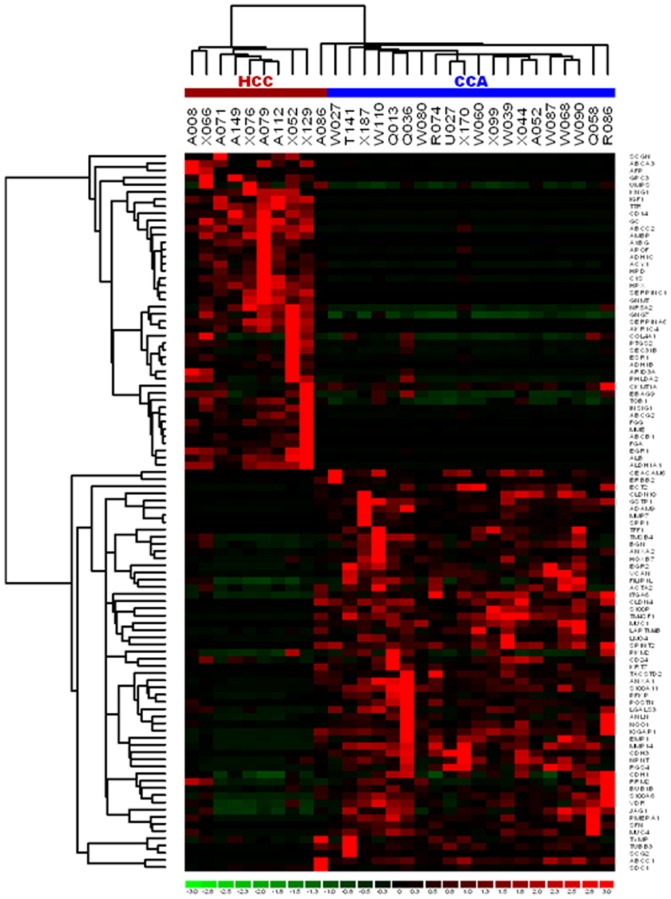
Unsupervised hierarchical clustering of CCAs and HCCs.

Using the in-house PCR array, we first examined the differential gene expression of tumor tissues from the CCA and HCC cases in the training set. An unsupervised hierarchical cluster analysis using the normalized gene expression data classified the 30 samples into two distinct groups (Fig. 1): one group contained nine HCCs and the other contained 20 CCAs and one HCC. There were 69 differentially expressed genes: 26 genes in the CCA cases and 43 genes in the HCC cases (Table 1). The overexpressed genes in the CCAs were associated with cell adhesion (e.g., *SPP1*, *MMP7* and *CLDN4*) and cell movement (e.g., *S100P*, *TMSB4* and *S100A11*). In contrast, the overexpressed genes in the HCCs were associated with xenobiotic metabolisms (*ADH1B*, *ADH1C* and *ALDH1A1*), biomolecule metabolisms (*APOF*, *DPYD* and *GC)*, cell proliferation (*IGF1* and *ARID3A*), differentiation (*EGR1* and *GPC3*) and the transport of small molecules (*ALB*, *AKR1C4* and *TTR*). The primer sequences of these genes are summarized in Table S3.

**Table 1:** List of top 20 overexpressed and 10 underexpressed genes in 20 CCA tissues.

No	UniGene ID	Symbol	Title	Mean*		*P* value
				CCAs	HCCs	
**Overexpressed genes**						
1	BG571732	*S100P*	S100 calcium binding protein P	2.2859	0.5445	0.0060
2	NM_001251830	*SPP1*	secreted phosphoprotein 1	1.7711	0.1281	0.0002
3	BQ688566	*MMP7*	matrix metallopeptidase 7 (matrilysin, uterine)	1.3315	0.0151	0.0003
4	BM923753	*TFF1*	trefoil factor 1	1.3116	0.0001	0.0001
5	BC023552	*SFN*	Stratifin	1.1101	0.1443	0.0017
6	BM926728	*GSTP1*	glutathione S-transferase pi1	0.9891	0.0630	<0.0001
7	BF680512	*TMSB4*	thymosin beta 4, X-linked	0.3832	0.2333	0.0453
8	BQ683841	*S100A11*	S100 calcium binding protein A11	0.2753	0.0469	0.0004
9	AK074480	*ANXA1*	annexin A1	0.1859	0.0361	0.0019
10	NM_001305	*CLDN4*	claudin 4	0.1632	0.0260	0.0009
11	NM_002483	*CEACAM6*	carcinoembryonic antigen-related cell adhesion molecule 6 (non-specific cross reacting antigen)	0.1330	0.0005	<0.0001
12	AL832780	*TM4SF1*	transmembrane 4 L six family member 1	0.0723	0.0072	0.0002
13	NM_003816	*ADAM9*	ADAM metallopeptidase domain 9	0.0721	0.0120	0.0045
14	NM_021102	*SPINT2*	serine peptidase inhibitor, Kunitz type, 2	0.0370	0.0045	<0.0001
15	X52228	*MUC1*	mucin 1, cell surface associated	0.0365	0.0006	<0.0001
16	NM_003870	*IQGAP1*	IQ motif containing GTPase activating protein 1	0.0308	0.0066	0.0002
17	AK128505	*KRT7*	keratin 7	0.0150	0.0018	0.0012
18	AK223249	*HOXB7*	homeobox B7	0.0057	0.0033	0.0453
19	BM904612	*S100A6*	S100 calcium binding protein A6	0.0013	0.0008	0.0165
20	NM_182848	*CLDN10*	claudin 10	0.0012	0.0000	0.0366
**Underexpressed genes**						
1	NM_000477	*ALB*	albumin	3.7085	71.6658	<0.0001
2	NM_021870	*FGG*	fibrinogen gamma chain	0.9397	20.2895	<0.0001
3	BE742013	*TTR*	transthyretin	0.2005	7.9316	<0.0001
4	NM_001904	*CTNNB1*	catenin (cadherin-associated protein), beta 1, 88kDa	1.7146	3.4423	0.0453
5	NM_001633	*AMBP*	alpha-1-microglobulin/bikunin precursor	0.0746	2.1106	<0.0001
6	AF130100	*SERPINC1*	serpin peptidase inhibitor, clade C (antithrombin), member 1	0.0414	1.8204	<0.0001
7	M58569	*FGA*	fibrinogen alpha chain	0.1090	1.8176	0.0001
8	NM_000689	*ALDH1A1*	aldehyde dehydrogenase 1 family, member A1	0.1674	1.7739	0.0001
9	NM_001164617	*GPC3*	glypican 3	0.0049	0.3247	0.0008
10	BC027881	*AFP*	alpha-fetoprotein	0.0002	0.2638	0.0002

*mean of expression level of individual gene after normalization with the geometric mean of *B2M* and *GAPDH* and transformed into a 2^∧^^-dCp^.

### Discrimination Analysis

**Figure 2:**
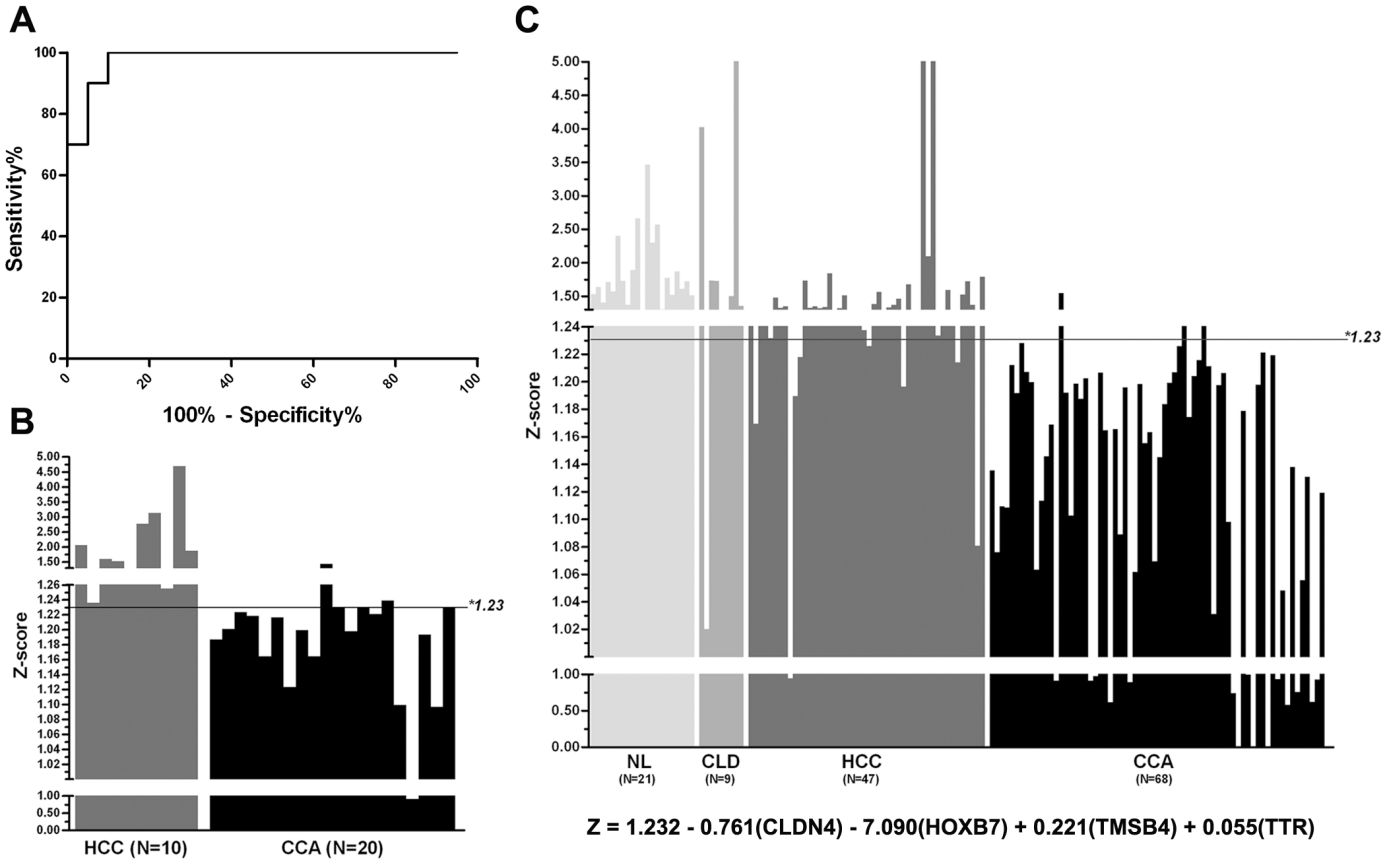
Discrimination analysis. From the predictive equation, the Z-score was calculated from the combination of CLDN4, HOXB7, TMSB4, and TTR. Using 1.23 as a cut-off value, most of CCAs can be distinguished from the others. (A) Predicting performance of Z-score using the ROC curve. (B) Results from the training set samples. (C) Results from the testing set samples. NL = non-cancerous liver tissue; CLD = chronic biliary-liver disease; HCC = hepatocellular carcinoma; CCA = cholangiocarcinoma.

The next goal was to identify the individual genes or combinations of genes which were related to diagnosis in the training set. A multiple linear regression analysis was used to find the best models composed of the fewest number of genes for use as a diagnostic equation for discriminating between CCA and HCC tissues. From the 69 differentially expressed genes obtained in the hierarchical cluster analysis, an equation involving a combination four genes, Z = 1.232−0.761(*CLDN4*)−7.09(*HOXB7*) +0.221(*TMSB4*)+0.055(*TTR*), gave the best discriminating power. In a receiver operating characteristic curve (ROC) analysis, this diagnostic equation yielded an area under the curve (AUC) of 0.98, and, when a Z-score of 1.23 was used as the optimal cut-off point to discriminate between CCA and HCC, the sensitivity and specificity were 90% and 100%, respectively (Figs. 2A–B). A Z-score of less than 1.23 indicated CCA rather than HCC. Hence the four-gene diagnostic equation was designated as “CCA diaganostic equation”.

### Validation of the “CCA diagnostic equation” in the testing set

To validate the accuracy of the “CCA diagnostic equation” in the discrimination of liver masses of CCA from HCC and noncancerous liver tissues, the expressions of *CLDN4*, *HOXB7*, *TMSB4* and *TTR* were verified in the larger testing set by real-time PCR. Using the Z-score of 1.23 as the cut-off point, the “CCA diagnostic equation” could distinguished CCA patients from non-CCA cases with a sensitivity of 95.6% and specificity of 88.3%, and the AUC was 0.94 (Fig. 2C). Finally, we sought to determine whether the “CCA diagnostic equation” developed in the current study was a more powerful discriminator than an individual gene marker or the serum markers routinely used for the diagnosis of CCA, namely carbohydrate antigen19-9 (CA19-9) and carcinoembrionic antigen (CEA). We first inspected the ROC analysis of the individual gene marker to obtain the best cut-off value from the training set, and then verified the diagnostic value in the testing set (n = 145). For serum markers, the diagnostic parameters were compared in the CCA, HCC and chronic biliary-liver cases whose serum CA19-9 and CEA were recorded (n = 66). As shown in Table 2, the “CCA diagnostic equation” gave better diagnostic results than a single gene marker. In addition, the equation also yielded a much better sensitivity, negative predictive value and false negative rate than the use of serum CA19-9 and CEA (Table 3).

**Table 2:** Comparison of diagnostic indices between each single marker and “CCA Diagnostic equation”.

Diagnostic indices (%)	Tissue mRNA marker (n = 145)				
	CLDN4	HOXB7	TMSB4	TTR	“CCA Diagnostic eq.”
Sensitivity	86.8	85.3	39.7	95.6	95.6
Specificity	72.7	53.2	70.1	79.2	88.3
Positive predictive value	73.8	61.7	54.0	80.2	87.8
Negative predictive value	86.2	80.4	56.8	95.3	95.8
Fault positive	14.5	24.8	15.9	11.0	6.2
Fault negative	6.2	6.9	28.3	2.1	2.1
AUC	0.87	0.77	0.56	0.91	0.94

AUC = area under curve of the receiver operating characteristic curve; CLDN4, claudin 4 (cut-off value was 0.01); HOXB7, homeobox B7 (cut-off value was 0.001); TMSB4, thymosin beta 4, X-linked (cut-off value was 0.08); TTR, transthyretin (cut-off value was 0.30); eq., equation.

**Table 3:** Comparison of diagnostic indices between serum markers and “CCA Diagnostic equation”.

Diagnostic indices (%)	Serum marker (n = 66)		
	CA19-9	CEA	“CCA Diagnostic eq.”
Sensitivity	44.4	38.9	97.2
Specificity	100.0	100.0	86.7
Positive predictive value	100.0	100.0	89.7
Negative predictive value	65.2	57.7	96.3
Fault positive	0.0	0.0	6.1
Fault negative	54.5	54.5	1.5
AUC	0.67	0.88	0.94

AUC = area under curve of the receiver operating characteristic curve; eq., equation; CA19-9, carbohydrate antigen 19-9 (cut-off value was 100 U/ml); CEA, carcinoembryonic antigen (cut-off value was 22 µg/ml.

### Validation by immunohistochemical staining

**Figure 3:**
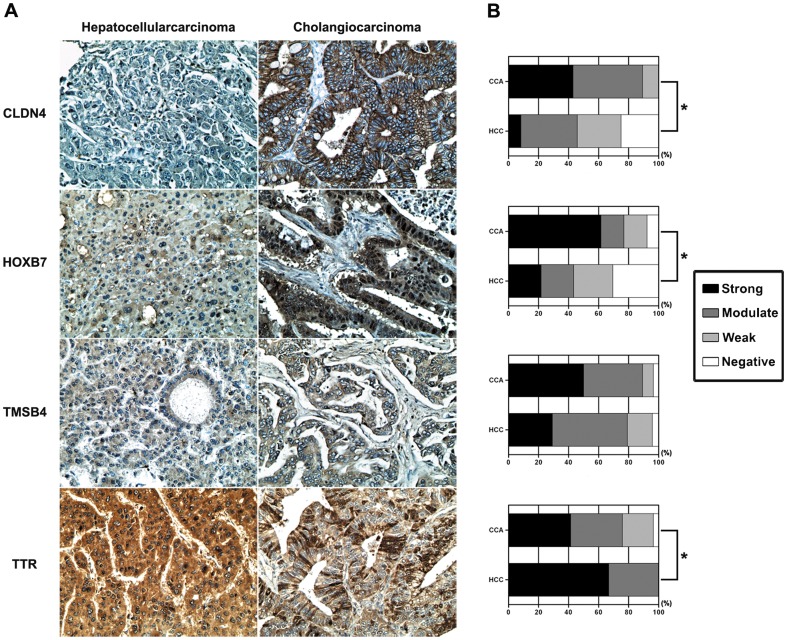
Immunohistochemistry of CLDN4, HOXB7, TMSB4, and TTR in tumor tissues from CCA and HCC patients. (A) CLDN4, HOXB7 and TMSB4 were obviously expressed in CCA tissues while TTR was strongly expressed in HCC tissues. (B) The expressions of CLDN4, HOXB7, TMSB4, and TTR were quantified based on the intensity. *P value<0.05. HCC = hepatocellular carcinoma; CCA = cholangiocarcinoma.

To establish that the four genes in the diagnostic equation reflected CCA and HCC tissue, we verified the expression levels of CLDN4, HOXB7, TMSB4 and TTR in the tumor tissues of CCA (n = 28) and HCC (n = 24) using immunohistochemistry. Compared with HCC, CCA expressed significantly higher levels of CLDN4 (*P*<0.001) and HOXB7 (*P*<0.01), a similar expression of TMSB4, but a lower level of TTR (*P*<0.02) (Fig. 3).

## Discussion

The accurate and definitive diagnosis of a tumor is necessary for a medical specialist to assess the severity of the disease and select the most suitable therapy. Histopathological diagnosis is the routine, standard method of diagnosing a solid tumor. However, there are some biopsy specimens and ill-defined tumor tissues which cannot be definitively diagnosed by general histopathology. Currently, the molecular signature characterized the malignant phenotype is usually reported. In this study, we developed and validated a model of gene expression which can distinguish liver tissues of CCA from HCC and benign biliary-liver diseases. A “CCA diagnostic equation” involving four genes (*CLDN4*, *HOXB7*, *TMSB4* and *TTR*) was formulated for the diagnosis of CCA with high sensitivity and specificity.

The unsupervised hierarchical cluster analysis retrieved from the in-house PCR array of the well-defined samples in the training set could initially separate patients into two groups: CCA and HCC cases. The results indicated that CCA cases share a characteristic gene expression profile which is distinguishable from HCC by a small subset of genes. The molecular functions and biological processes of the overexpressed genes in CCA were involved in the regulation of cell adhesion and migration. Conversely, the overexpressed genes in HCC were associated with xenobiotic and biomolecule metabolisms. Our PCR array profiling data are consistent with those reported using SAGE and oligonucleotide microarray analyses which specified the upregulation of genes associated with cell adhesion molecules in CCA [14], [15].

The gene expression profiling results have also yielded lists of genes which are potential biomarkers for diagnosis. We first identified the best models for discriminating CCA from HCC in the training set using multiple linear regression analysis. After extensive cross-validation, a combination of *CLDN4*, *HOXB7*, *TMSB4* and *TTR* designated as “CCA diagnostic equation”, yielded the best equation for differentiating CCA from HCC (Fig. 2, Table 2). In our experience, majority of the advanced hilar CCAs always invade the liver parenchyma and form large focal liver mass undistinguishable from ICC and HCC. In this study, the liver masses from hilar CCAs were included in both training set and testing set. Regardless to CCA origin, the “CCA diagnostic equation” can differentiate the tumor masses of ICC and hilar CCA from HCC.

The immunohistochemistry of the four diagnostic genes expressed in CCA and HCC tissues revealed a higher expression of CLDN4, HOXB7 in CCA tissues and a higher expression of TTR in HCC tissues. The formulated equation was further validated in the testing set with a larger sample size and a variety of non-CCA controls. Sensitivity (the true positive rate) and specificity (the true negative rate) are statistical measures of a binary classification test performance. A perfect predictor would be described as 100% sensitivity and 100% specificity which can be represented graphically as a ROC with AUC = 1. In the present study, the AUC of each test demonstrated that the “CCA diagnostic equation” could effectively diagnose CCA cases from the controls with a higher sensitivity and specificity compared with those of individual gene analysis. The use of the “CCA diagnostic equation” was also superior to the use of known serum markers; CA19-9 and CEA as the “CCA diagnostic equation” gave the highest AUC compared to serum those of CA19-9 and CEA. Since no correlation was found between these serum markers and the “CCA diagnostic equation” (data not shown), the inclusion of serum markers and molecular markers “CCA diagnostic equation” may increase its diagnostic power, and this needs to be investigated.

The better diagnostic parameters obtained from the “CCA diagnostic equation” formulated in the present study are consistent with the finding of a previous study [15] in which a different diagnostic equation was reported to improve both the sensitivity and specificity of the diagnosis of ICC when compared with the use of CA19-9 and CEA. A comparison of the gene expression profiles in parasite-associated (Thai patients) and non-parasite-associated (Japanese patients) human ICCs demonstrated different molecular signatures between the two sample groups [13]. An elevated expression of genes involved in xenobiotic metabolism was found in the parasite-associated ICCs whereas genes related to growth factor signaling were shown in the non-parasite-associated ICCs. These findings may explain the difference in the set of genes formulated for the diagnostic equation in our study and that reported by Nishino [15]. As a consequence, one should aware of possible limitation of our study, namely that the vast majority of CCA cases in this study are likely to have been associated with parasitic infection and may not fully reflect the various risk factors responsible for the cholangiocyte neoplastic transformation.

CLDN4 is a transmembrane protein which is critical for the conformation and function of tight junctions. In our study, the expression of *CLDN4* in CCA tissues was approximately 6-fold greater than in HCC tissues and hence it is probably be a marker of CCA. Similar observation was reported in ICC [15]. Recently, many investigators have paid attention to CLDN4 as it is the specific receptor of *Clostridium perfringens* enterotoxin (CPE) [17], which causes a loss of osmotic equilibrium and subsequent cell death via apoptosis or oncosis mediated by Ca^2+^-influx [18]. For this reason, CLDN4 has been proposed as a target molecule for cancer therapy.

HOXB7, a member of the homeobox family, encodes a protein with a homeobox DNA-binding domain and functions as a sequence-specific transcription factor which is involved in cell proliferation and differentiation [19]. The overexpression of *HOXB7* in CCA tissues observed in the current study is supported by previous reports [12], [13]. In addition, *HOXB7* has been suggested as a marker in brush cytology specimens to distinguish bile duct cancer patients from patients with biliary strictures [20].

TMSB4 is an actin sequestering protein which is involved in the regulation of actin polymerization and many other functions, such as cell migration [21], differentiation and angiogenesis [22]. In this study, expression of *TMSB4* was 1.6-fold higher in CCA tissues than HCC. Over-expressions of TMSB4 in osteosarcoma, esophagus cancer, and colorectal cancer have been reported [23]. A close association between the overexpression of TMSB4 and enhanced tumor-cell invasion has been demonstrated in colorectal cancer [24].

TTR or pre-albumin encodes transthyretin which acts as a transporter protein for thyroid hormones and retinol (vitamin A) in the plasma [25]. TTR is synthesized mainly in hepatic tissue, therefore, a decrease in plasma TTR was reported in cases of severe liver diseases, malnutrition and acute inflammation [26]. In the current study, the average expression of *TTR* in CCA tissues was significantly lower than that of HCC. This may support the lower level of TTR in serum of CCA patients compared to those suffering from benign hepatobiliary diseases and healthy controls [27]. Similar observations have been reported in sera of patients with ovarian cancer, advanced cervical and endometrial carcinomas [28]. The mechanism by which TTR is reduced in cancer is still unknown.

In summary, the definite diagnosis of a tumor is necessary for effective treatment of CCA. At present, serodiagnosis is the general approach, but it provides unsatisfactory sensitivity and specificity. This study offers a new formula for improving the accuracy of diagnosis of CCA in a region where this type of cancer is primarily associated with a parasitic infection. Further validation is needed to confirm the expression of the four genes used in the formula in a larger cohort and in CCA patients with other types of growth pattern, such as the periductal infiltrating and intraductal growth types. An additional challenge is to explore the possibility of using the “CCA diagnostic equation” to diagnose the non-liver fluke CCA from other countries.

## Supporting Information

Table S1 **Clinicopathological features of samples used for training set.**
(DOC)

Table S2 **Clinicopathological features of samples used for testing set.**
(DOC)

Table S3 **Primer sequences data.**
(DOC)
