# Supplementary material for: A Novel Predictive Equation for Potential Diagnosis of Cholangiocarcinoma
Source: PLoS One. 2014 Feb 28;9(2):e89337. doi: 10.1371/journal.pone.0089337 (PMC3938437; doi:10.1371/journal.pone.0089337)
Supplement: Table S1 — Clinicopathological features of samples used for training set. (DOC) [file pone.0089337.s001.doc]

**Table S1 Clinicopathological features of samples used for training set**

| **CCA** | **N = 20** |  | **HCC** | **N = 10** |
| --- | --- | --- | --- | --- |
| **Gender** |  |  | **Gender** |  |
| Male | 11 |  | Male | 8 |
| Female | 9 |  | Female | 2 |
| **Age** |  |  | **Age** |  |
| < 56 | 6 |  | < 56 | 4 |
| ≥ 56 | 14 |  | ≥ 56 | 6 |
| **Anatomical gross type** |  |  | **Histopathological type** |  |
| ICC | 16 |  | Broad trabecular | 4 |
| ECC | 4 |  | Clear cell | 1 |
| **Histopathological type** |  |  | Trabecular | 2 |
| Papillary | 10 |  | Mixed type | 3 |
| Non-papillary | 10 |  |  |  |
| **Staging** |  |  | **Staging** |  |
| I – II | 7 |  | I – II | 8 |
| III – IV | 13 |  | III – IV | 2 |
