# Supplementary material for: A Novel Predictive Equation for Potential Diagnosis of Cholangiocarcinoma
Source: PLoS One. 2014 Feb 28;9(2):e89337. doi: 10.1371/journal.pone.0089337 (PMC3938437; doi:10.1371/journal.pone.0089337)
Supplement: Table S2 — Clinicopathological features of samples used for testing set. (DOC) [file pone.0089337.s002.doc]

**Table S2 Clinicopathological features of samples used for testing set**

| **CCA** | **N = 68** |  | **HCC** | **N = 47** |
| --- | --- | --- | --- | --- |
| **Gender** |  |  | **Gender** |  |
| Male | 42 |  | Male | 37 |
| Female | 26 |  | Female | 10 |
| **Age** |  |  | **Age** |  |
| < 56 | 23 |  | < 56 | 29 |
| ≥ 56 | 45 |  | ≥ 56 | 18 |
| **Anatomical gross type** |  |  | **Histopathological type** |  |
| ICC | 49 |  | Broad trabecular | 12 |
| ECC | 19 |  | Clear cell | 2 |
| **Histopathological type** |  |  | Trabecular | 16 |
| Papillary | 19 |  | Mixed type | 17 |
| Non-papillary | 48 |  |  |  |
| Mixed type | 1 |  |  |  |
| **Staging** |  |  | **Staging** |  |
| I – II | 17 |  | I – II | 32 |
| III – IV | 41 |  | III – IV | 7 |
| NA | 10 |  | NA | 8 |

NA = not applicable
