# Supplementary material for: A Novel Predictive Equation for Potential Diagnosis of Cholangiocarcinoma
Source: PLoS One. 2014 Feb 28;9(2):e89337. doi: 10.1371/journal.pone.0089337 (PMC3938437; doi:10.1371/journal.pone.0089337)
Supplement: Table S3 — Primer sequences data. (DOC) [file pone.0089337.s003.doc]

Table S3 Primer sequences data

| **No** | **Symbol** | **Primer sequence** | |
| --- | --- | --- | --- |
|  |  | **Forward** | **Reverse** |
| ***26 overexpressed genes in CCA*** | | |  |
| 1 | *ABCC1* | 5'-CTGGGCTTATTTCGGATCAA-3' | 5'-TGAATGGGTCCAGGTTCATT-3' |
| 2 | *ADAM9* | 5'-GCACAAGAACCACAATTAAGATG-3' | 5'-GCGAGTAACTGCGTGAATAC-3' |
| 3 | *ANXA1* | 5'-AACGCTTTGCTTTCTCTTG-3' | 5'-CTTCTGGTGGTAAGGATGG-3' |
| 4 | *CEACAM6* | 5'-CAGCCGTGTGTTCAAATG-3' | 5'-AGACTCCTCCCTGTTCAG-3' |
| 5 | *CLDN10* | 5'-GCTCAATGACTGGATGTTC-3' | 5'-CCGTTGTATGTGTATCTGG-3' |
| 6 | *CLDN4* | 5'-TACATTTTCCCCACTCTGTC-3' | 5'-CAAACCTGTTTACAGCACCT-3' |
| 7 | *GSTP1* | 5'-GATCTCCTTCGCTGACTAC-3' | 5'-TGTTTCCCGTTGCCATTG-3' |
| 8 | *HOXB7* | 5'-CTGTGGGTCTGGACTAAC-3' | 5'-GGGACGGGAAGCAAGAAGC-3' |
| 9 | *IQGAP1* | 5'-AAAGACACCCAAGAAGCACAGAAG-3' | 5'-GACTCCATACAAGCCAACATCAGG-3' |
| 10 | *ITGA6* | 5'-TCCTCCAGATAGTGTAGC-3' | 5'-GCATAGTCTTGTGATGTG-3' |
| 11 | *KRT7* | 5'-CTGAAGGCTTATTCCATCC-3' | 5'-ACTGTCTCACTGTCTTGG-3' |
| 12 | *MMP14* | 5'-TCTTCGTTGCTCAGTCAG-3' | 5'-CATCCAAGGCTAACATTCG-3' |
| 13 | *MMP7* | 5'-GATAAGCACTGTTCCTCCAC-3' | 5'-CACAGTCACACCATAAAGGA-3' |
| 14 | *MUC1* | 5'-CGTAGCCCCTATGAGAAG-3' | 5'-CTGAGTGGAGTGGAATGG-3' |
| 15 | *S100A11* | 5'-ATGATGAAGAAACTGGACAC-3' | 5'-AAAGGCTGGAAGGAAAGG-3' |
| 16 | *S100A6* | 5'-GGGGAGACTCGTCACCAG-3' | 5'-GCTTACACAACGCACATTCG-3' |
| 17 | *S100P* | 5'-AAGGTGCTGATGGAGAAGG-3' | 5'-GCCACGAACACGATGAAC-3' |
| 18 | *SFN* | 5'-CCTTGTGGCTGAGAACTG-3' | 5'-CCTCAATCTCGGTCTTGC-3' |
| 19 | *SPINT2* | 5'-CTGACCAAGGAGGAGTGC-3' | 5'-TTGAACATATCGCTGGAGTG-3' |
| 20 | *SPP1* | 5'-GCGAGGAGTTGAATGGTG-3' | 5'-ATAATCTGGACTGCTTGTGG-3' |
| 21 | *TACSTD2* | 5'-GATGCCGCCTACTACTTCG-3' | 5'-TCGTCCAGGTAATAGATGAGC-3' |
| 22 | *TFF1* | 5'-CCAGAAGAGGAGTGTGAATTTTAG-3' | 5'-TAGTCAAAGTCAGAGCAGTCAATC-3' |
| 23 | *TM4SF1* | 5'-CTGGCTACTGTGTCATTG-3' | 5'-ACAGAGATACATTCCATTCC-3' |
| 24 | *TMSB4* | 5'-GACTTCGCTCGTACTCGT-3' | 5'-CTCTGTCTTCTTCAGTTTCGA-3' |
| 25 | *TUBB3* | 5'-GGATCAGCGTCTACTACAAC-3' | 5'-TGAAGAGATGTCCAAAGGC-3' |

Table S3 Primer sequences data (cont.)

| **No** | **Symbol** | **Primer sequence** | |
| --- | --- | --- | --- |
|  |  | **Forward** | **Reverse** |
| ***26 overexpressed genes in CCA (cont.)*** | | |  |
| 26 | *VCAN* | 5'-GGCACCTGTTATCCTACTG-3' | 5'-GTCTCGGTATCTTGCTCAC-3' |
| ***43 underexpressed genes in CCA*** | | |  |
| 1 | *A1BG* | 5'-CTACACCTGCCGCTACCG-3' | 5'-CGTCTCATCGCTCAGAATCAG-3' |
| 2 | *ABCA3* | 5'-AGAAATACGGTGCCGGCTATCACA-3' | 5'-CAATGCCCAGCTCTTTCTGCTTCT-3' |
| 3 | *ABCB1* | 5'-AGAGACATCATCAAGTGGAGAG-3' | 5'-AGCAAGGCAGTCAGTTACAG-3' |
| 4 | *ABCC2* | 5'-TCGGAATGTGAATAGCCTGAAG-3' | 5'-CGCAAGGATGATGAAGAATATCG-3' |
| 5 | *ACY1* | 5'-CCTCCGTGAACCTGACTAAG-3' | 5'-GCATCCACTTCTGAGCAAAC-3' |
| 6 | *ADH1B* | 5'-AAAGTGCTTCATTGACTAAACAG-3' | 5'-TTTGTGCCATTTCTTAGTTACC-3' |
| 7 | *ADH1C* | 5'-ATTGATGGGAAATTATTTG-3' | 5'-TATTGGCTTCAATTCCCCAGT-3' |
| 8 | *AFP* | 5'-AAAGCCCACTCCAGCATCG-3' | 5'-GCAGCCCAAAGAAGAATTGTAGG-3' |
| 9 | *AKR1C4* | 5'-AAGAAGTGGCAAGCAATGG-3' | 5'-AGAATCAATATGGCGGAAGC-3' |
| 10 | *ALB* | 5'-CGCTATTAGTTCGTTACACCAAG-3' | 5'-GTTCAGGACCACGGATAGATAG-3' |
| 11 | *ALDH1A1* | 5'-GCTTCTTTCCCTTAGTGACTC-3' | 5'-TGACAAGCAGACATGACATCC-3' |
| 12 | *AMBP* | 5'-GTCCACACCAACTATGATG-3' | 5'-AACCACTCTGAAGTCCTG-3' |
| 13 | *APOF* | 5'-TCCTCATCCAGCATCTTC-3' | 5'-CTTCTCATTCTCACAGTCC-3' |
| 14 | *ARID3A* | 5'-CCGAAGGCTCTGGTGTCTG-3' | 5'-GGGAGTGGTGAGTGTGTGG-3' |
| 15 | *C1S* | 5'-TGACTGGATAATGAAGACTATG-3' | 5'-ATAAGGAACAGAAGGTAATGC-3' |
| 16 | *CD14* | 5'-CCTCAATCTGTCGTTCGC-3' | 5'-GTCCAGTGTCAGGTTATCC-3' |
| 17 | *COL7A1* | 5'-GCTTAATGTGGTGATGTTG-3' | 5'-CTGAGTAGTGAAGGATGC-3' |
| 18 | *CTNNB1* | 5'-GTATGGGTAGGGTAAATCAGTAAG-3' | 5'-GAAGCATCGTATCACAGCAG-3' |
| 19 | *DPYD* | 5'-TTGGTGTTGGATAGAGGATAGG-3' | 5'-CAGTCAGAGCCCGTATGTG-3' |
| 20 | *EBAG9* | 5'-TCAGTTCCTAAGCAGACAGATG-3' | 5'-ATAGTCAGGTTCCAGTTGTTCC-3' |
| 21 | *EGR1* | 5'-GCTGTCACCAACTCCTTC-3' | 5'-CATCTCCTCCTCCTGTCC-3' |
| 22 | *ESR1* | 5'-CCTGCCACGGTCTGAGAG-3' | 5'-ATGTGCCACTAAGAACTGAGC-3' |
| 23 | *FGA* | 5'-ATTCCCTTCCCGTGGTAAATC-3' | 5'-TCTCTTGGTGCTATGTGTTCC-3' |

**Table S3 Primer sequences data (cont.)**

| **No** | **Symbol** | **Primer sequence** | |
| --- | --- | --- | --- |
|  |  | **Forward** | **Reverse** |
| ***43 underexpressed genes in CCA (cont.)*** | | |  |
| 24 | *FGFR2* | 5'GATAACAACACGCCTCTC-3' | 5'-TCAATTCCCACTGCTTCC-3' |
| 25 | *FGG* | 5'-ACTCCTAATGGTTATGATAATGGC-3' | 5'-GGTGTTGCTGTCCTTCTCC-3' |
| 26 | *GC* | 5'-CAGGTCAGCCAACTTGTG-3' | 5'-TGGTGTCATAGCAGTCAGG-3' |
| 27 | *GNG7* | 5'-AGACGGCGAGACCTCCTG-3' | 5'-GGCTATGTTGTTAGTGGCTGAC-3' |
| 28 | *GNMT* | 5'-CTGGCTTGAGTAAGTTCC-3' | 5'-TTGTAAGGCTTGAAGTCG-3' |
| 29 | *GPC3* | 5'-GCCGAATGCTCACCAGAATG-3' | 5'-TCCACCACACCTGCCATAC-3' |
| 30 | *HPD* | 5'-ATTGTGTTTGTCCTCTCC-3' | 5'-AACTTGTCTTGCTCTACC-3' |
| 31 | *HPX* | 5'-ACCGTGGAGAATGTCAAG-3' | 5'-CCTGGAAGCAGTAGTAGC-3' |
| 32 | *IGF1* | 5'-CTCTTCAGTTCGTGTGTG-3' | 5'-CATCTCCAGCCTCCTTAG-3' |
| 33 | *INSIG1* | 5'-GAGGTGGAGAATGATGAC-3' | 5'-GGTTGGTAATTGAGTCTTAG-3' |
| 34 | *KNG1* | 5'-GCATAACTGAAGCCACTAAG-3' | 5'-TAGCCACGGAGAATTTCG-3' |
| 35 | *METAP2* | 5'-GATGTTCGTCTGTGTGATGTTG-3' | 5'-TCCTTCCTCCATTCTTGTTGC-3' |
| 36 | *NR5A2* | 5'-TTAATCACCTCTAATACTCATCC-3' | 5'-CTGCTGTTCTCTGTAATGC-3' |
| 37 | *POU1F1* | 5'-TTCATTATTCTGTTCCTTCCTGTC-3' | 5'-CGCCTGAGTTCCTGCTTG-3' |
| 38 | *PTGS2* | 5'-TCAGACAGCAAAGCCTAC-3' | 5'-CCACACTCATACATACACC-3' |
| 39 | *RGS5* | 5'-CCTCCTCCTCCTCCTCTC-3' | 5'-TTCTACATCCTCAATTCACTCC-3' |
| 40 | *SCGN* | 5'-GCTTGCTGGTATGTTCTTATCTG-3' | 5'-AGGTCTCGGAGGAAGTTGC-3' |
| 41 | *SERPINA6* | 5'-TGAACCCAGTGTAAGAGAC-3' | 5'-GAAGAACTTGGAGGAGATTG-3' |
| 42 | *SERPINC1* | 5'-AACTGAACTGCCGACTCTATC-3' | 5'-CGCTCTGGATTGCTCTGC-3' |
| 43 | *TTR* | 5'-ATTCTTGGCAGGATGGCTTC-3' | 5'-CAGAGGACACTTGGATTCACC-3' |
| ***2 internal control genes*** | | |  |
| 1 | *B2M* | 5'-AAGATGAGTATGCCTGCCG-3' | 5'-CGGCATCTTCAAACCTCC-3' |
| 2 | *GAPDH* | 5'-CGCTCTCTGCTCCTCCTG-3' | 5'-ACTCCGACCTTCACCTTCC-3' |
